# Genome-wide functional analysis of human 5' untranslated region introns

**DOI:** 10.1186/gb-2010-11-3-r29

**Published:** 2010-03-11

**Authors:** Can Cenik, Adnan Derti, Joseph C Mellor, Gabriel F Berriz, Frederick P Roth

**Affiliations:** 1 Harvard Medical School, Department of Biological Chemistry and Molecular Pharmacology, 250 Longwood Avenue, SGMB-322, Boston, MA 02115, USA; 2 Center for Cancer Systems Biology, Dana Farber Cancer Institute, 44 Binney Street, Boston, MA 02115, USA

## Abstract

**Background:**

Approximately 35% of human genes contain introns within the 5' untranslated region (UTR). Introns in 5'UTRs differ from those in coding regions and 3'UTRs with respect to nucleotide composition, length distribution and density. Despite their presumed impact on gene regulation, the evolution and possible functions of 5'UTR introns remain largely unexplored.

**Results:**

We performed a genome-scale computational analysis of 5'UTR introns in humans. We discovered that the most highly expressed genes tended to have short 5'UTR introns rather than having long 5'UTR introns or lacking 5'UTR introns entirely. Although we found no correlation in 5'UTR intron presence or length with variance in expression across tissues, which might have indicated a broad role in expression-regulation, we observed an uneven distribution of 5'UTR introns amongst genes in specific functional categories. In particular, genes with regulatory roles were surprisingly enriched in having 5'UTR introns. Finally, we analyzed the evolution of 5'UTR introns in non-receptor protein tyrosine kinases (NRTK), and identified a conserved DNA motif enriched within the 5'UTR introns of human NRTKs.

**Conclusions:**

Our results suggest that human 5'UTR introns enhance the expression of some genes in a length-dependent manner. While many 5'UTR introns are likely to be evolving neutrally, their relationship with gene expression and overrepresentation among regulatory genes, taken together, suggest that complex evolutionary forces are acting on this distinct class of introns.

## Abstract 2

Genes with short 5'UTR introns have higher expression than genes with no or long 5'UTR introns. Complex evolutionary forces act on these introns.

## Background

The advent, evolution and functional significance of introns in eukaryotes have been topics of intense debate over the past 30 years (reviewed in [1,2]). There are two major opposing views on when introns arose in evolution; this 'introns-early' versus 'introns-late' controversy is reviewed in [1,2]. Also, debate exists on what causes their frequent losses and gains [3,4] and whether they have any adaptive significance.

Neutral or nearly neutral population genetic processes under general, non-adaptive conditions have been suggested to result in dynamic gains and losses of introns. Such neutral processes could account for some of the observed patterns of intron presence [5], but do not rule out the possibility that adaptive processes are simultaneously contributing to the maintenance of some introns. Introns have been suggested to confer adaptive advantages by functioning in diverse mechanisms ranging from modifying recombination rates to increasing the efficacy of natural selection [6,7], and even to protecting exons from deleterious R-loops [8]. A relatively well-understood functional role of introns is to facilitate the production of distinct forms of mature mRNA through alternative splicing [9-12]. Recent genome-wide analyses suggest that nearly 95% of all human genes are alternatively spliced [13-15]. Many alternative splicing events are tissue-specific, and functional regulatory elements in exons and introns are associated with tissue specificity of these variants [16,17]. Therefore, introns can contribute to gene regulation.

Most of the theoretical and empirical work on the evolution of introns has focused on those found in coding regions, yet an appreciable fraction of human genes (approximately 35%) contain introns in their 5'UTRs [18]. Introns in 5'UTRs are twice as long as those in coding regions, on average, and moderately lower in density, such that 5'UTRs contain a lower percentage of intronic bases than do coding regions [19]. By contrast, 3'UTRs are typically much longer than 5'UTRs but a study in human, mouse, fruit fly and mustard weed have shown that relatively few 3'UTRs (<5%) contain introns [19]. This observation is partly explained by nonsense-mediated decay given that an intron downstream of the stop codon would typically signal a transcript for degradation by nonsense-mediated decay [20,21]. In addition, splicing signals within 3'UTRs have been suggested to have reduced maintaining selection and, therefore, 3'UTRs tend to be longer and contain fewer introns compared to 5'UTRs [22]. In summary, these differences suggest that introns in different regions of genes constitute distinct functional classes with unique evolutionary histories.

As 5'UTR introns (5UIs) are unusually long and can considerably increase the total number of bases transcribed for a given gene, it is useful to consider the two main adaptationist theories about the functional consequences of intron length. The first model argues that it is energetically costly for cells to transcribe long stretches of DNA that does not encode protein [23]. By this reasoning, total intronic length should be relatively low in highly expressed genes. Consistent with this prediction, the most highly expressed genes tend to have shorter introns in both humans and the worm *Caenorhabditis elegans *[23], and there seems to be additional selective pressures towards having shorter proteins and more biased codon usage [24,25]. However, an opposite effect is observed in *Oryza *and *Arabidopsis*, such that highly expressed genes have more and longer introns [26]. If the selection against longer introns in highly expressed genes minimizes the energetic cost of unnecessary transcription, this observation is unexpected, as we would expect the model to hold across all taxa.

The second model, termed 'genome design', posits that the pressure to maintain many intronic regulatory elements favors longer introns in tissue-specific genes [27]. The main supporting observation for this hypothesis is that human 'housekeeping' genes tend to be compact, with fewer and shorter introns as well as shorter coding regions relative to tissue-specific genes [28,29]. Tissue-specific genes, on the other hand, tend to have longer and more conserved introns, perhaps because their functional complexity requires a more stringent level of regulation [30]. Furthermore, genes with higher functional complexity tend to be longer and seem to be under more complex regulation [27]. However, analyses of human antisense genes contradict the claims of the genome design hypothesis [31,32]. These studies showed that antisense genes, which need to be expressed rapidly, are compact but can be tissue-specific regulators [31,32]. Curiously, some studies supporting the genome design hypothesis explicitly disregard 5UIs (see methods in [27]) even though these introns might be expected to include regulatory elements, being closer to transcription and often to translation start sites [33,34].

Neither of these two principal theories addresses the possible role of 5UIs and the evolutionary pressures acting on them; therefore, the functional significance, if any, of their frequent occurrence remains unclear. Given that splicing of these sequences seemingly has no effect on the amino acid sequence of the encoded protein, it is unclear what selective benefit might accompany their removal from the mature mRNA. The reduced splice-site conservation and high variability in length of 5UIs have led to the suggestion that they contract and expand without significant functional consequences [19]. However, an exception to the trend of reduced splice-site conservation is observed in *Cryptococcus*, an intron-rich fungus with longer 5' and 3' UTR introns than coding region introns [35] and high conservation near UTR intron boundaries [36].

Given these conflicting results and the scarcity of studies regarding the evolution of UTR introns, it is worthwhile to consider a functional perspective. An analysis of functional trends among human genes with 5UIs could lead to a better understanding of their evolution and also potentially to the detection of novel mechanisms of regulation mediated by these introns. Here, we analyze expression profiles of genes with 5UIs and examine the distribution of these introns in different functional categories of genes.

## Results

### Characterization of a set of genes with 5'UTR introns

**Figure 1:**
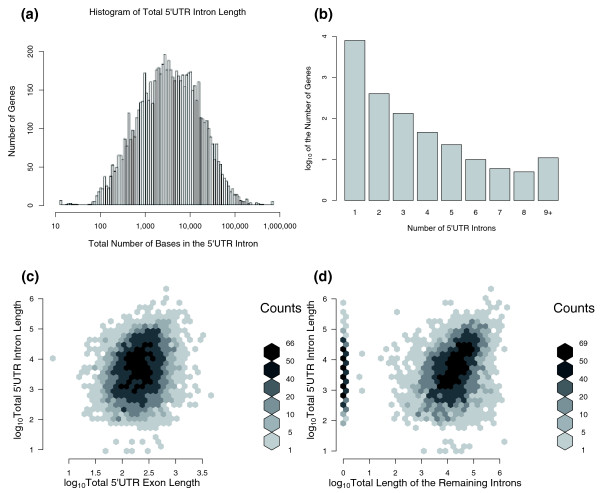
**Characterization of fundamental properties of 5'UTR introns**. **(a) **Histogram of the total 5'UTR intron length. A well annotated set of RefSeq transcript IDs are used in this analysis and this histogram shows the distribution of the log_10 _of the total number of intronic nucleotides in the 5'UTR. **(b) **Distribution of the number of introns in the 5'UTR. The log_10 _of number of transcripts that have a given number of introns in their 5'UTR is shown. The number of transcripts with a given number of 5'UTR introns decreases exponentially. **(c) **Heat map depicting the relationship between total lengths of 5'UTR introns and 5'UTR exons. **(d) **Heat map depicting the relationship between total lengths of 5'UTR introns and non-5'UTR introns. In both heatmaps, darker shades of gray indicate more transcripts.

To investigate the functional properties of human 5UIs, we used NCBI's Reference Sequence (RefSeq) collection. These are curated, full-length sequences with annotated UTR boundaries, and expression data are available for many of them. The lack of a translation reading frame makes the computational prediction of splice sites in 5'UTRs inherently more difficult [37], necessitating the choice of such a validated set. In humans, approximately 8.5k (35%) out of 24.5k RefSeq mRNAs contained at least one intron in their 5'UTR (Additional file 1). Previous estimates of the percentage of genes with 5UIs ranged between 22% and 26% [18] and 38% [19] in humans, suggesting that the RefSeq collection had no major bias in terms of presence or absence of 5UIs compared to other previously used datasets. The distribution of total 5'UTR intronic length for genes in our dataset was also similar to that observed previously (Figure 1a). The inter-quartile range of total length of 5UIs within each gene was approximately 1.3 - 16 kb. Some 5UIs were extremely long -- 16% were longer than 27 kb, the length of the average protein coding gene in the human genome [38], and 5% were longer than 76 kb (Figure 1a). As previously reported [18,19], most genes had few 5UIs. More than 90% had a single intron, and the percentage of genes with two or more introns decreased exponentially (Figure 1b).

We next considered the relationship between the total lengths of 5'UTR exons and of 5UIs. Even though there was a correlation between the lengths of 5UIs and 5'UTR exons overall, this correlation was slight and was driven by the genes with the longest 5UIs (Figure 1c; Pearson correlation coefficient or Pearson correlation coefficient (PCC) = 0.21, *P *< 2.2e-16). In fact, when genes with 5UI lengths in the lowest 25th percentile were analyzed, the correlation was no longer significant (Figure 1c; PCC = -0.005, *P *= 0.84). A statistically significant, albeit slight, correlation was found for genes with 5UI length below the median (Figure 1c; PCC = 0.07, *P *= 8.4e-05). Among the genes with 5UIs, a similar relationship was evident between the total length of 5UIs and the total length of the remaining introns (Figure 1d). Although these two variables were significantly correlated (Figure 1d; PCC = 0.18, *P *< 2.2e-16), the relationship was clearly driven by the genes with longer 5UIs. When genes with 5UI lengths either in the lowest 25th or 50th percentile were considered, correlation was negligible (Figure 1d; PCC = -0.02 and 0.04, *P *= 0.53 and 0.04, respectively).

Thus, genes with long 5UIs tend to have a high total intronic length and longer 5'UTR exons. While this tendency holds in genes with additional introns, several genes with total 5UI lengths greater than 10 kb lack any coding-region or 3'UTR introns (Figure 1d). On the other hand, amongst genes with short 5UIs, the total length of 5UIs is uncorrelated with the lengths of either 5'UTR exons or the remaining introns.

### Gene expression analysis

**Figure 2:**
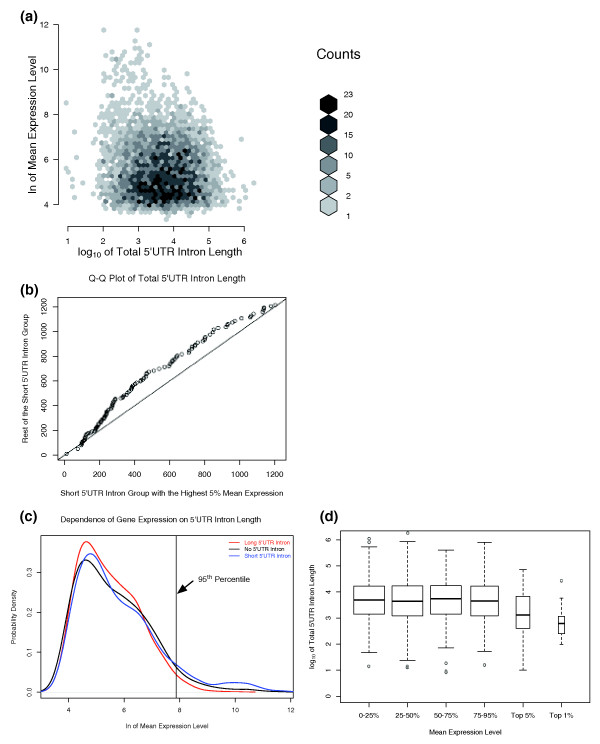
**Expression analysis as a function of total 5'UTR intron length**. **(a) **Heat map of the mean expression level versus the total 5'UTR intron length. The shade of gray represents the number of transcripts in each bin with darker shades implying more transcripts. The overrepresentation of short 5'UTR-intron-containing genes among the highest expression levels is apparent. **(b) **Quantile-quantile plot of total 5'UTR intron length of short 5'UTR intron-containing genes divided into highly expressed (top 5%) and other genes. The most highly expressed genes tend to have shorter 5'UTR introns. **(c) **Smoothed histogram of the mean expression level with respect to presence/absence of 5'UTR intron and its length. A kernel density estimator was fitted to the expression data and the corresponding probability density is plotted as a function of the mean expression level. The black line corresponds to the probability density for transcripts without any 5'UTR introns. Genes with long 5'UTR introns are represented by the red line while genes with short 5'UTR introns are represented by the blue line. The vertical line represents the top 5% of mean expression level of all genes. **(d) **Total 5'UTR intron length of genes in different expression level categories. The width of the boxes represents the relative number of data points in each category. Transcripts in the top 1% and top 5% in expression level tend to have shorter 5'UTR introns.

We next examined gene expression-related predictions of the two principal models of intron evolution. Previous studies have suggested that the genes with the highest expression levels are selected to have shorter introns [23]. If a similar selective pressure were acting on 5UIs (in conjunction with neutral evolutionary processes [19]), one would expect a tendency towards reduced gene expression level as a function of increased 5UI length in a subset of genes. We therefore compared gene expression from 79 tissues as a function of the total 5'UTR intronic length. We divided 5UI-containing genes into three categories with respect to the total 5'UTR intronic length (short, 0 to 25%; intermediate, 25 to 75%; long, 75 to 100% in length). The short 5UI-containing genes were highly overrepresented in the top 1% of mean expression level for the genes with 5UIs (Fisher's exact test, *P *= 3.3e-15) and also in the top 5% (Fisher's exact test, *P *= 1.7e-14) (Figure 2a). These genes were 12.7 times more likely than all other genes with 5UIs to be in the highest 1% of mean expression and 3 times more likely to be in the highest 5% of mean expression. There was also a global trend for genes with short 5UIs to be expressed at a higher level compared to genes with longer 5UIs (25 to 100 percentile in length; one-sided Wilcoxon rank sum test, *P *= 2.98e-05; Figure 2a).

The enrichment for high expression in genes with short 5UIs held even when genes with the longest 25% of 5UIs were removed. In this case, the genes with the highest 1% and 5% expression were, respectively, 9.5 times and 2.5 times more likely to have short 5UIs as opposed to intermediate length 5UIs (25 to 75 percentile in length; Fisher's exact test, *P *= 1.53e-11 and *P *= 3.21e-10, respectively).

The most highly expressed 5UI-bearing genes show a striking tendency to harbor short 5UIs. Of all 5UI-containing genes, 26% had a total 5UI length below 1.3 kb. By contrast, the corresponding fractions for genes in the top 5% and 1% by expression were 50% and 83%, respectively. We then separated short 5UI-containing genes into two groups: the most highly expressed genes (top 5% in expression); and the remaining genes. For the most highly expressed genes, the inter-quartile range of total 5UI length was 215 to 734 nucleotides compared with 289 to 870 nucleotides for the remaining genes (Figure 2b). Thus, the most highly expressed genes in humans are very strongly enriched for short 5UIs.

Interestingly, no expression dependence was observed among genes with intermediate or long 5UIs: genes with long 5UIs (top 25th percentile in length) did not tend to be expressed less than those with the intermediate length 5UIs (Wilcoxon rank sum test, *P *= 0.25). Also, no statistically significant depletion for the long 5UI category was observed in either the top 1% or the top 5% expression group (Fisher's exact test, *P *= 0.29, odds ratio = 0.25, and *P *= 0.017, odds ratio = 0.58, respectively). Thus, we did not observe the inverse relationship between expression and total 5UI length that might have been expected under the energetic cost model.

Next, we considered all RefSeq genes and asked whether having an intron in the 5'UTR has an effect on overall expression. We found no differences in 5UI representation in the top 1% or the top 5% of the mean expression groups. Furthermore, no difference was detected in the distribution of mean expression between genes with and without 5UIs (two-sided Wilcoxon rank sum test, *P *= 0.17). However, genes with short 5UIs were 1.8 times more likely to be in the top 5% and 3.3 times more likely to be in the top 1% in overall expression level than genes with no 5UIs (Fisher's Exact Test, *P *= 3.15e-08 and *P *= 7.57e-07, respectively) than genes with no 5UIs (Figure 2c). Thus, the presence of short 5UIs is correlated with high mean expression.

The observed expression trends could reflect the influence of genomic features other than 5UIs. Yet, short 5UIs do not seem to predict a short total length of either non-5'UTR introns or 5'UTR exons (Figure 1c, d). Furthermore, when genes in the top 5% in mean expression were divided into two groups with respect to 5UI presence or absence, we observed no differences in total non-5'UTR intron length between genes with 5UIs and those that lack these introns (Wilcoxon rank sum test, *P *= 0.20, data not shown). Therefore, the tendency of highly expressed genes to have short 5UIs is unlikely to be confounded by the effects of 5'UTR exons or the remaining introns.

For genes with the highest expression levels, these results are in contrast to the neutral model of 5UI evolution, which predicts that 5'UTR intronic length should not depend on expression level. These results are also not explained by the energetic cost hypothesis, which would predict that genes with the highest expression levels should be less likely to have 5UIs. In stark contrast to the predictions of each model, we found the most highly expressed genes to be significantly enriched in short 5UIs. Furthermore, the energetic cost hypothesis would also predict a linear decrease in the total 5UI length as a function of increasing gene expression. Yet, we found no overall differences with respect to 5UI length except for the most highly expressed genes. Even though a neutral model of 5UI evolution is plausible for most genes, our results for the most highly expressed genes are inconsistent with both neutral and energetic cost models (Figure 2d).

We next used expression to assess the applicability to 5UIs of the other major hypothesis of intron evolution, the 'genome design model', which predicts that intermediate or long introns should be enriched in tissue-specific genes as a consequence of complex regulation. As originally outlined, the genome design model explicitly disregards 5UIs [27]; however, a direct corollary of this hypothesis is that genes with higher variance in expression across tissues should have intermediate or long introns in their 5'UTRs as well.

**Figure 3:**
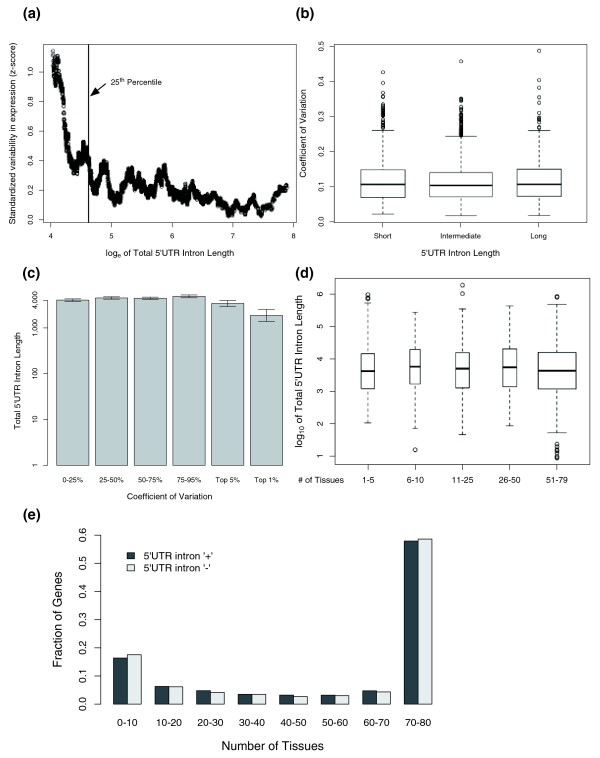
**Analysis of variability in expression across tissues as a function of the total 5'UTR intron length**. **(a) **Transcripts with low mean expression have higher normalized expression variability. A standardized measure of the variability in gene expression across tissues was calculated and plotted against the natural logarithm of mean expression level. The black vertical line represents the lowest 25th percentile in mean expression. Since transcripts with low levels of mean expression tend to exhibit an artificially high variability in expression, they are removed from further analysis. **(b) **Boxplot of the coefficient of variation (standard deviation-to-mean ratio) of genes grouped by the total length of 5'UTR intron. The width of the boxes represents the relative number of data points in each category. There are no apparent differences between the three groups **(c) **Boxplot of log_10 _of total 5'UTR intron length of genes grouped by their across-tissue variability. Genes are divided into six categories depending on their coefficient of variation. Error bars correspond to standard deviation of the mean. No obvious dependence of expression variability to total 5UI length can be observed except for the most highly variable genes, which tend to have slightly shorter 5'UTR introns. **(d) **Boxplot of log_10 _of total 5'UTR intron length for gene groups defined by the number of tissues in which expression of each gene was detected. A gene was defined to have detectable expression in a given tissues if its expression was higher than the 25th percentile of mean expression of all genes. We found no differences in total 5'UTR intron length amongst the different gene groups. **(e) **Histogram of number of genes divided by the presence of 5'UTR introns and by the number of tissues in which expression was detected. The number of tissues in which expression was detected was independent of the presence of 5'UTR introns.

We sought to address two potential sources of bias. First, gene expression levels vary greatly and variance is strongly correlated with mean expression. Therefore, we calculated the standard deviation-to-mean ratio (coefficient of variation or CV) [39], a normalized measure of dispersion, for each gene across all tissues. Second, due to technological limitations of expression arrays, precise measurement of expression level is more difficult for genes with low or no expression in a given tissue; therefore, artificially high variance in expression might be observed for genes with low mean expression across all tissues. We therefore calculated a robust measure of dispersion that minimizes this effect:

where *CV*_*x *_is the CV of expression of gene *x *across all tissues, ***y***_*x *_represents the vector of CV values for all 201 genes in a window centered around gene *x*, while *μ*_1/2 _and *MAD *represent the median and median absolute deviation, respectively. As expected, genes with low expression tended to have much more variability across tissues (Figure 3a). Based on the observed trend line, the genes with the lowest 25% expression were removed from further analysis (Figure 3a). The remaining genes were sorted into three categories with respect to the total intronic 5'UTR length as before (short, 0 to 25%; intermediate, 25 to 75%; long, 75 to 100%). We found no significant differences between these groups with respect to inter-tissue variability as measured by the coefficient of variation (Figure 3b; Kruskal-Wallis rank sum test, df = 2, *P *= 0.23). We then examined the lengths of the introns as a function of variability in expression (Figure 3c). The genes with the highest 5% variability across tissues did not differ from the other genes with respect to their 5UI lengths (Wilcoxon rank sum test, *P *= 0.07, 95% confidence interval between -0.008 and 0.25), but the genes with highest 1% across-tissue variability tended to have slightly shorter 5UIs (Wilcoxon rank sum test, *P *= 0.006, 95% confidence interval between -0.67 and -0.11). Genes with short 5UIs were also overrepresented in the top 1% across-tissue variability category (Fisher's Exact Test, *P *= 0.005, odds-ratio = 2.7). Our results suggested that length of the 5UI was not a major factor in determining across-tissue variability but there was a preference for shorter 5UIs in the most variable genes.

Although our approach reliably captures across-tissue variability in gene expression, it disregards any potential effects of 5UI presence or length on how widely a gene is expressed. To consider the potential impact of such effects, we calculated the number of tissues in which expression was detected for each gene. Based on our analysis presented in Figure 3a, we defined a given gene as 'present' in a given tissue if its expression was greater than the 25th percentile in the distribution of mean expression over all tissues, calculated for all genes. Genes were placed into one of five classes according to the number of tissues in which they were present. No significant difference was detected amongst the corresponding five distributions of total 5UI length (Figure 3d; Kruskal-Wallis rank sum test, df = 4, *P *= 0.19). Furthermore, the distribution of number of tissues in which each gene was present did not differ between genes containing and lacking 5UIs (Figure 3e). These results clearly contradict predictions of the 'genome design' hypothesis, in that narrowly expressed genes did not show a greater tendency to contain 5UIs nor did they tend to have longer 5UIs. These results strongly suggest that the evolution of 5UIs is not driven primarily by the selective pressures proposed by the 'genome design' hypothesis.

### Functional enrichment of Gene Ontology categories

Under the neutral model, genes with 5UIs should be uniformly distributed across functional groups. We used Gene Ontology (GO) function annotations to determine which groups of genes are enriched or depleted in 5UIs, if any. Two popular functional trend analysis tools, FuncAssociate [40] and GoStat [41], were used for this analysis. One key challenge was the translation of the gene identifiers from RefSeq RNA IDs to those used in the GO database. There are different approaches to this problem and the two software packages differ from each other in this respect. FuncAssociate uses the Synergizer [42] software to resolve the problem of synonyms while GoStat uses definitions in the UniGene database as well as the information provided in the GO databases. Both software packages yielded very similar results, suggesting that our general conclusions were independent of the methods of synonym resolution or enrichment calculation.

A significant overrepresentation of genes with 5UIs was found in many regulatory pathways (Table 1). Non-receptor protein tyrosine kinases (NRTKs) formed the most highly overrepresented group, followed by genes involved in the regulation of actin organization, transcriptional regulators, and zinc ion binding proteins (Table 1). NRTKs lack transmembrane domains and therefore do not recognize extracellular ligands, unlike the majority of protein tyrosine kinases. Nevertheless, they play crucial roles in nearly all aspects of biology and are implicated in many cancers (reviewed in [43]). Among NRTKs, genes harboring 5UIs encode key regulatory kinases, such as the proto-oncogene tyrosine kinase *SRC*, c-src tyrosine kinase (*CSK*), janus kinases (*JAK*), spleen tyrosine kinase (*SYK*), tec protein tyrosine kinase (*TEC*), and Bruton agammaglobulinemia tyrosine kinase (*BTK*) among others.

**Table 1:** Overrepresented Gene Ontology attributes for genes with 5'UTR introns

*N*	*X*	*LOD*	*P*	*P-adj*	Gene Ontology attribute	
25	35	0.650	1.4e-05	0.0153	GO:0004715:	non-membrane spanning protein tyrosine kinase activity
27	38	0.644	7.5e-06	0.0073	GO:0051261:	protein depolymerization
31	44	0.633	2.1e-06	0.0017	GO:0051494:	negative regulation of cytoskeleton organization and biogenesis
32	48	0.560	9.2e-06	0.0085	GO:0032956:	regulation of actin cytoskeleton organization and biogenesis
32	49	0.534	1.8e-05	0.0193	GO:0032970:	regulation of actin filament-based process
48	76	0.497	6.6e-07	0.0004	GO:0051493:	regulation of cytoskeleton organization and biogenesis
39	62	0.491	8.3e-06	0.0078	GO:0016459:	myosin complex
43	71	0.449	1.2e-05	0.0120	GO:0051129:	negative regulation of cellular component organization and biogenesis
51	88	0.404	1.1e-05	0.0114	GO:0033043:	regulation of organelle organization and biogenesis
105	216	0.243	3.5e-05	0.0398	GO:0015629:	actin cytoskeleton
1094	2356	0.232	5.7e-33	<0.0001	GO:0008270:	zinc ion binding
139	294	0.220	1.3e-05	0.0139	GO:0003779:	actin binding
996	2218	0.199	1.4e-23	<0.0001	GO:0006355:	regulation of transcription, DNA-dependent
1000	2233	0.197	3.4e-23	<0.0001	GO:0051252:	regulation of RNA metabolic process
1061	2380	0.195	7.5e-24	<0.0001	GO:0045449:	regulation of transcription
1013	2273	0.193	1.2e-22	<0.0001	GO:0006351:	transcription, DNA-dependent
1015	2277	0.193	9.5e-23	<0.0001	GO:0032774:	RNA biosynthetic process
191	420	0.190	8.3e-06	0.0077	GO:0008092:	cytoskeletal protein binding
1078	2436	0.189	6.6e-23	<0.0001	GO:0019219:	regulation of nucleobase, nucleoside, nucleotide and nucleic acid metabolic process
1106	2512	0.185	1.3e-22	<0.0001	GO:0010468:	regulation of gene expression
1189	2713	0.183	1.6e-23	<0.0001	GO:0031323:	regulation of cellular metabolic process
1088	2477	0.182	8.6e-22	<0.0001	GO:0006350:	transcription
1211	2791	0.175	4.7e-22	<0.0001	GO:0019222:	regulation of metabolic process
989	2267	0.174	1.2e-18	<0.0001	GO:0003677:	DNA binding
1507	3515	0.172	2.9e-25	<0.0001	GO:0003676:	nucleic acid binding
1212	2825	0.165	5.5e-20	<0.0001	GO:0046914:	transition metal ion binding
1682	4053	0.147	1e-20	<0.0001	GO:0050794:	regulation of cellular process
1157	2784	0.136	5.6e-14	<0.0001	GO:0016070:	RNA metabolic process
1758	4305	0.134	3.7e-18	<0.0001	GO:0050789:	regulation of biological process
1772	4364	0.129	4.2e-17	<0.0001	GO:0005634:	nucleus
1463	3584	0.127	1.1e-14	<0.0001	GO:0006139:	nucleobase, nucleoside, nucleotide and nucleic acid metabolic process

*N *represents the number of transcripts in the RefSeq collection that have both a 5'UTR intron and a given GO attribute; *X *represents the total number of transcripts having that GO attribute. For each attribute, *P *is the nominal *P*-value obtained from a one-tailed Fisher's Exact Test that calculates the probability that at least *N *transcripts have the particular attribute given the number of genes with 5'UTR introns. This nominal *P*-value is adjusted for multiple hypothesis testing to yield *P-adj *using a resampling approach that accounts for dependencies among the tested hypotheses (see [40] for precise procedure). The table is sorted in descending order by the log_10 _of the odds ratio (*LOD *score), where  and *M *is the number of all genes, *e *is a pseudocount of 0.5 and *q *is the query set size. All attributes with *LOD *> 0.125 and a *P-adj *< 0.05 are reported.

To gain insight into the evolution of NRTK 5UIs, we identified orthologous genes in mouse and rat genomes corresponding to each human NRTK. We collected 5'UTR features for these genes in each genome using RefSeq annotations (Additional file 2). More widely studied organisms tend to have more accurate transcript structures and include many more splice variants in the RefSeq collection. For example, 18 human genes were represented by more than one transcript, while only four mouse and no rat NRTKs had more than one splice variant. The paucity of transcripts in some mammalian species is more likely to have arisen from limited testing rather than biology, given recent studies suggesting that alternative splicing is ubiquitous across several taxa [9].

UTRs are also generally less well defined in less intensively studied organisms. For example, *ABL2*, *BTK*, *FRK *and *SRC *all lack defined 5'UTR boundaries in the rat RefSeq collection, even though EST evidence suggests that *SRC*, *BTK *and *ABL2 *all have 5'UTR-containing transcripts (data not shown). Another current limitation is ambiguity in identifying the specific branch in which a given deletion or insertion event took place. Despite these shortcomings, a comparison of orthologs already provides insight into the dynamics of the evolution of 5UIs in NRTK genes.

**Figure 4:**
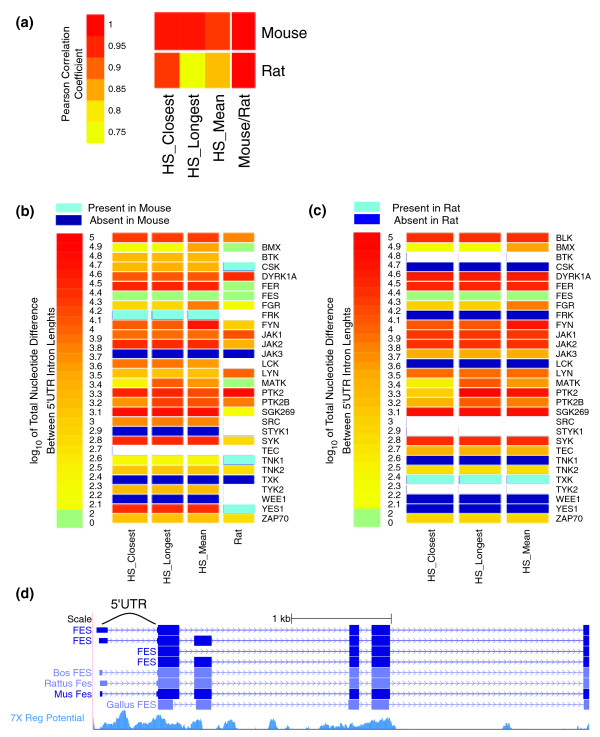
**Comparative genomics of 5'UTR introns within non-receptor tyrosine kinases**. Several human NRTKs have multiple splice isoforms and for these we used three different methods for calculating total 5'UTR intron length: mean of 5'UTR intron length for isoforms with 5'UTR introns (HS_Mean); longest total 5'UTR intron length (HS_Longest); 5'UTR intron length most similar to its ortholog in the genome of interest (HS_Closest). **(a) **Heatmap of length correlation (considering genes with non-zero 5'UTR intron lengths) was plotted for the specified comparisons. As expected from the evolutionary distances between the analyzed species, the highest correlation (93%) was observed between mouse and rat NRTKs. **(b) **For each mouse ortholog of a human NRTK, the heatmap depicts the changes in total 5'UTR intron length (color reflects log_10 _of total 5'UTR intron length). The histogram above the color scale summarizes the distribution of changes in 5'UTR intron length. A 5'UTR intron may be present in mouse but not in the compared species (light blue) or vice versa (dark blue). Comparisons require an annotated 5'UTR for each ortholog, and were therefore not possible in some cases (white). **(c) **Same as (b) but substituting 'rat' for 'mouse'. **(d) **Human genomic region containing the 5'UTR and first few coding exons (UCSC Genome Browser view). '7X Regulatory Potential', for which higher scores indicate a greater potential for harboring regulatory sequence elements, was calculated using alignments of seven mammalian genomes as previously described [44].

When every ortholog of a given NRTK had at least one annotated 5UI, the lengths of those introns were generally highly correlated (Figure 4a). Given the number of different splice variants for each human gene, we used three different approaches to calculate the 5UI length for each gene. We either used the mean length of splice variants with non-zero 5UI lengths, or picked the variant with the longest 5UIs, or the one whose length was closest to its ortholog in either of the rat or mouse genomes. All three measures resulted in high correlation overall between 5UI lengths across species (PCC ranged between 89 and 91% for human-mouse and 79 and 89% for human-rat comparisons; *P *< 0.0001 for all; Figure 4a). As expected from evolutionary distances, the highest correlation in 5UI lengths was observed between rat and mouse orthologs of NRTKs (PCC = 93%, *P *= 1.4e-07).

Despite a generally strong correlation in 5UI length among orthologs, some sets of orthologs had a widespread distribution of length changes. While the total 5UI length of *FES *changed by less than five nucleotides in all possible comparisons, rat *PTK2 *and mouse *PTK2 *5UIs differed by approximately 63.5 kb (Figure 4b, c). The length conservation observed for the *FES *5UI is notably consistent with the high regulatory potential previously calculated for this 5UI [44] (Figure 4d). More broadly, introns containing regulatory regions might be expected to have high length conservation.

When each orthologous group of NRTKs was analyzed, we found variability with respect to presence/absence of 5UIs in some of these groups. For example, *STYK1 *and *WEE1 *both had 5UIs in humans, but not in mouse or rat (Figure 4b, c). In the case of human *WEE1*, two transcripts were identified in the human RefSeq collection - while one variant had a 512-nucleotide 5UI, the other variant lacked 5UIs entirely. This observation suggested the possibility that intron-containing variants might be present in mouse and rat without being represented in the RefSeq transcript collection. Indeed, we found EST evidence that rat *WEE1 *has a splice variant that includes a 5UI [GenBank:CK603528.1]. On the other hand, mouse *FRK *(Figure 4b) and rat *TXK *(Figure 4c) had 5UIs while their orthologs did not. We also observed several NRTKs having 5UIs in two of the species but not in the other one. For example, both human and mouse orthologs of *LCK*, *BTK*, *CSK*, *TNK1*, and *YES1 *had annotated 5UIs, while both human and rat orthologs of *JAK3 *and *TEC *had annotated 5UIs (Figure 4b, c). Our results suggest that NRTK 5UIs are frequently conserved, a conclusion that would be further strengthened should the apparent gain/loss events be attributable to incomplete transcript annotation.

**Figure 5:**
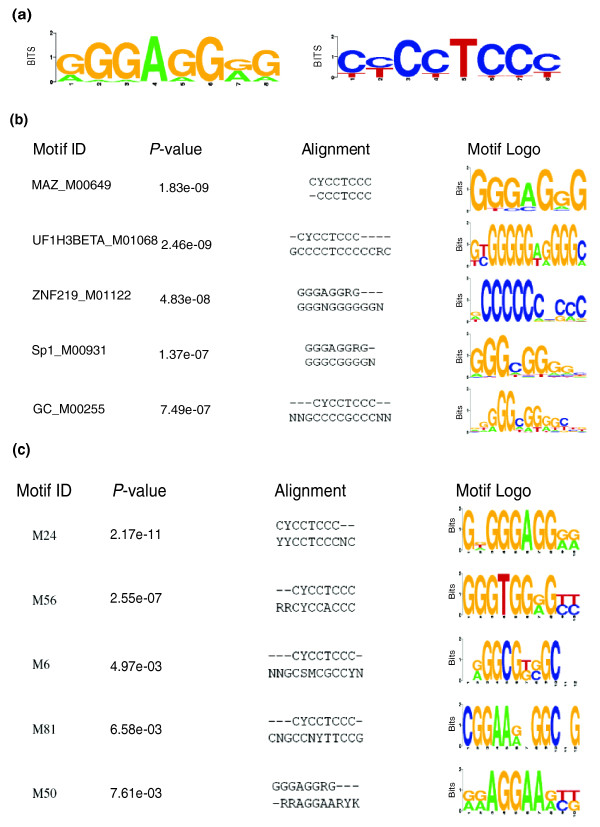
**Characterization of an 8-nucleotide DNA motif in the 5'UTR of human NRTKs**. **(a) **Representative motif and its reverse complement. **(b) **Comparison of the representative motif to the TRANSFAC v11.3 database of known transcription factor binding sites. **(c) **Comparison of the representative motif to a list of conserved human predicted motifs [46]. STAMP website was used for the comparisons [47]. The default ungapped Smith-Waterman alignment was used and the *P*-value was calculated using the methods of Sandelin and Wasserman [74].

The appearance of 5UIs in most human NRTKs (Table 1) suggested the potential for a common regulatory mechanism acting via shared motifs. To search for shared and conserved motifs in these introns, human NRTK 5UI sequences were located in human-to-mouse and human-to-rat genome alignments. For 37 out of 42 human NRTKs, more than 10% of the 5UIs could be aligned to both genomes; only these conserved fragments were used for motif finding. Overrepresented RNA and DNA motifs were sought in these aligned sequences using the PhyloGibbs software [45]. In our search for overrepresented RNA elements, we identified two complementary motifs, so that the motif in these 5UIs is more likely to be relevant at the DNA level. A representative DNA motif (Figure 5a) with the highest log-posterior-probability was compared to the TRANSFAC v11.3 database of known transcription factor binding sites and to a list of conserved human predicted motifs [46] using the STAMP website [47] (Figure 5b, c). In both comparisons, the known binding site motif of the MAZ transcription factor was the most likely match. However, this does not rule out the possibility of this motif being the target of another DNA binding protein.

### Comparison between 5'UTR and 5'-proximal coding introns

5UIs are, by definition, the most 5'-proximal introns in their transcript. However, not all 5'-proximal introns need lie within the 5'UTR. We sought to understand whether the observed functional properties of 5UIs were shared with 5'-proximal coding region introns (5PCIs). Given that the median position of the first 5UI was approximately 130 nucleotides away from the transcription start site regardless of the number of 5UIs [19], we defined the genes without a 5UI but with a coding region intron within 150 nucleotides of the transcription start site as 5PCI-containing genes. This criterion resulted in 24% of 5UI-lacking genes having a coding region intron that was deemed to be a 5PCI.

We next used GO annotations to compare the functional properties of 5UI-lacking genes with 5PCIs to those without 5PCIs. We observed the strongest enrichment of 5PCIs among genes in the following functional groups: MHC protein complex 1, cytosolic ribosome, hemoglobin complex, glutathione transferase activity, and transmembrane transporters (Additional file 3). This result contrasts the observed enrichment of 5UIs in regulatory genes. The differences in the enrichment profiles suggest that distinct functional groups of genes prefer early introns in either the 5'UTR or the coding region but not in both.

**Figure 6:**
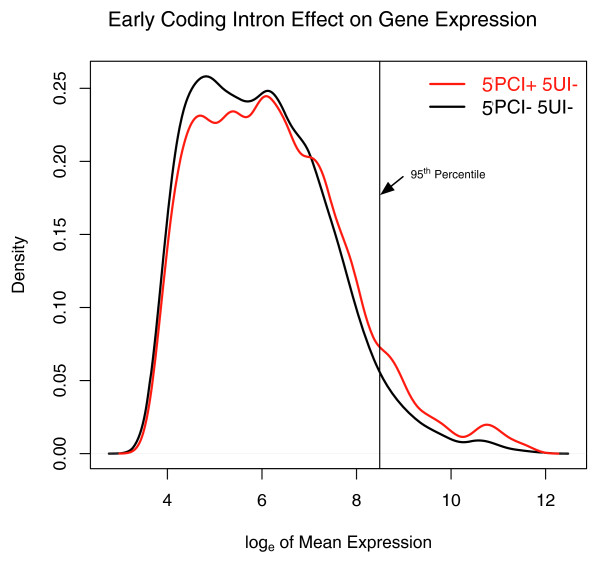
**The effect of 5'-proximal coding intron presence on gene expression**. **(a) **Smoothed histogram of the mean expression level with respect to presence/absence of 5'-proximal coding region introns (5PCIs). A kernel density estimator was fitted to the expression data and the corresponding probability density is plotted as a function of the mean expression level. The black line corresponds to the probability density for transcripts without any 5'UTR introns or any 5PCIs. The red line represents the probability density for 5'UTR intronless transcripts that have 5PCIs. The vertical line represents the top 5% of mean expression level of all genes without 5'UTR introns.

To assess the possible effect of 5' proximity on gene expression, we analyzed microarray data from the human gene expression atlas for 5UI-lacking genes. We found that genes with 5PCIs were more highly expressed on average (one-sided Wilcoxon rank sum test, *P *= 6e-08; Figure 6). We also observed a 2.3- and 3.7-fold enrichment for genes with 5PCIs among the most highly expressed top 5% and 1% of genes, respectively (Fisher's Exact Test, *P *= 4e-15 and *P *= 4e-09, respectively; Figure 6). The correlation between high expression and 5PCI presence was evident without any consideration of these introns' lengths. In contrast, no expression difference was observed between genes with or without 5UIs, on average, but short 5UIs were highly enriched among the most highly expressed genes (Figure 2c). These results suggest that early introns (both 5PCIs and 5UIs) are associated with the most highly expressed genes, but that this correlation is limited to short introns for 5UIs.

## Discussion

We compared the expression patterns and functional annotations of genes with and without 5UIs. We found that the most highly expressed genes reveal a strong enrichment for having short 5UIs as opposed to having either no 5UIs or longer 5UIs. This effect was specific to genes with the highest expression levels and no relationship between length and expression level was observed for genes with intermediate or long introns (Figure2d). These results are contrary to the energetic cost model [23], which predicts that genes with no 5UIs will be more highly represented among those with the highest expression levels. Because expression reflects both production and degradation rates of mRNAs, our results suggest that short 5UIs tend to either enhance transcription or stabilize mature mRNAs.

The prevalence and the significance of these intron-dependent mechanisms of transcriptional enhancement at a genome-wide level are poorly understood in mammalian systems. There are a few examples in mammals of increased transcription due to the proximity of an intron to the transcription start site [48-52], and these can be divided into two major categories with respect to the mechanism of enhanced transcription. The first mechanism is at the DNA level and involves the presence of activating transcription regulatory elements in the intron or the modulation of nucleosome positioning to make the promoter more accessible [52]. Similarly, 5UIs and other 5'-proximal introns in plants were shown to enhance gene expression at the transcriptional level in a position-specific manner [53,54]. The second mechanism is at the mRNA level, obviously related to splicing. *In vitro *studies have linked position-specific splicing and transcription enhancement mechanistically by demonstrating a direct interaction between the spliceosomal U small nuclear ribonucleoproteins with transcription elongation factors [55].

Our study thus suggests a distinction between 5UIs and 5PCIs with respect to their effects on gene expression. A splicing-dependent explanation might be the most compatible with the overall higher expression of genes with early coding-region introns compared to those without such introns. In contrast, even though a splicing-dependent effect may exist for 5UIs as well, the most highly expressed genes are highly enriched in having short 5UIs (approximately less than 1 kb in length), but 5UI presence or absence alone (without considering 5UI length) does not correlate with gene expression. Therefore, for 5UIs, short intron length seems to be a more important predictor of a high expression level than the presence or absence of 5UIs.

Given the inconsistency between our observations and the energetic cost hypothesis, we suggest two alternative models of 5UIs' effect on gene expression. The first model is that splicing-dependent enhancement in gene expression is influenced not only by the position of an intron, but also its size. The second model is that transcriptional regulatory proteins are recruited as a result of the presence of DNA elements, which in turn enhance expression level. This process could be restricted spatially, such that if the distance between the regulatory element and the transcription start site is long, then the enhancement should be less pronounced. Hence the genes with the highest expression levels might be under selective pressure to keep their introns short in order to retain their enhancer elements closer to the transcription start site. In this scenario, one can further imagine these elements to function in a tissue-specific regulatory mechanism if the recruited factors are themselves tissue-specific. Such an enhancer, located in the first intron of the mammalian acetylcholinesterase gene, was previously found to mediate the tissue-specific expression of this gene [56]. Another example of tissue-specific gene expression enhancement mediated by a 5UI was reported for the rice gene *rubi3 *[57].

The pressure to maintain regulatory elements in introns is also the central idea of the genome design model, and we tested the applicability of this hypothesis to 5UIs by analyzing genes with tissue-dependent variability in gene expression. As the most proximal intron to the transcription start site has been shown to contain more regulatory elements [33,34], the genome design model might be expected to apply to 5UIs as well as coding region introns. Specifically, the genome design hypothesis predicts that tissue-specific or highly variable genes contain many regulatory elements in their introns and hence have longer introns in general [30]. However, we found no relationship between variability in expression across tissues and the length of the 5UI (Figure 3a, b). Furthermore, neither 5'UTR presence nor length was correlated with how widely a gene was expressed. Most known nucleotide-level regulatory elements are short (<15 nucleotides), and most known *cis*-regulatory modules could be contained within even a short (<1 kb) 5UI. Therefore, 5UIs need not be particularly long to enable complex and conserved regulation via *cis*-regulatory elements. Our results support the idea that the genome design model is not likely to be the most useful guide for understanding the evolved lengths of 5UIs.

Finally, we considered whether certain classes of genes preferentially include 5UIs, and whether 5UIs contain regulatory elements. We found that genes with regulatory functions are enriched for 5UIs. The non-receptor tyrosine kinases, which play fundamental roles in all aspects of cell biology and signal transduction, were the most strongly enriched gene category. We identified a conserved DNA motif in the 5UIs of many non-receptor tyrosine kinases that could function by recruiting transcription factors. This recruitment might lead to tissue- or condition-specific regulation of NRTKs. For example, in the gene encoding Bruton's tyrosine kinase (a non-receptor tyrosine kinase), an SP1 transcription factor binding site was identified within the 5UI [58]. Furthermore, a point mutation in the 5UI region was shown to be associated with X-linked agammaglobulinemia, suggesting a functional role for this intron [58].

It is worth considering other forms of selection pressure that might affect 5'UTRs and therefore 5UIs. Upstream AUGs (uAUGs) tend to decrease translational efficiency, so that highly expressed genes should tend to avoid uAUGs in exons. On the other hand, intronic uAUGs are spliced out before the mature message encounters the cytoplasmic translation machinery; hence, they should not have a similar effect. The negative selection pressure against exonic uAUGs that tends to favor increased intronic sequence content within 5'UTRs [19] should be expected to be most pronounced for the most highly expressed genes. Our observation that the most highly expressed genes are enriched in having short 5UIs runs contrary to this expectation. Furthermore, shorter 5UIs did not imply shorter 5'UTR exon lengths, which might complicate our expectation for uAUG effects. Thus, models based solely on uAUG-based selection cannot explain the overrepresentation of short 5UIs among the most highly expressed genes.

Alternative splicing has emerged as a fundamental mechanism of regulation and expansion of the proteome, with nearly 95% of all genes thought to be alternatively spliced in mammals [13-15]. Tissue-dependent alternative splicing within 5'UTRs is common and can be functionally important. For example, aberrant splicing of 5'UTRs of *BRCA1 *and *ERβ *was recently implicated in carcinogenesis [59]. Whether these different splice variants play any regulatory role is unknown in all but a few cases. A plausible mechanism for the potential impact of alternative splicing in 5'UTRs is an effect on translation efficiency through differential inclusion of uAUGs.

The functional importance of alternative splicing in 5'UTRs is exemplified by human *NOD2*, which is associated with Crohn's disease. Only a subset of *NOD2*'s multiple splice variants include the uAUGs in the mature mRNA, and these have decreased translation efficiency [60]. Alternative splicing of 5'UTRs can also affect mRNA secondary structure. In the ETS domain transcription factor *ELK1*, for example, a facultative secondary structure modulates translation initiation [61]. Yet another connection between splicing and translation is the deposition of the exon junction complex following splicing, which induces translation through an interaction with the mammalian target of rapamycin (mTOR) signaling pathway [62]. The position or the sequence composition of the intron could potentially affect this splicing-dependent enhancement of translation efficiency by the mTOR pathway. These mechanisms of additional regulation by alternative splicing of 5UIs may underlie our observation that these introns are enriched in regulatory genes. Given that regulatory genes must themselves be precisely governed, additional means of regulation may allow for greater control, flexibility or complexity. Future work will need to address the full genome-wide functional implications and importance of alternative splicing of 5UIs.

## Conclusions

Our results highlight the functional importance of 5'UTR introns. Existing models predicting selective effects, such as avoidance of uAUGs, minimization of transcriptional cost, or accumulation of regulatory elements, do not suffice to explain results from our genome-scale analysis of 5UIs. Given 5UI enrichment and depletion in specific functional categories of genes, and the potential ability of 5UIs to enhance gene expression, a complex interplay of multiple selective forces appears to have influenced the evolution of this distinct class of introns.

## Materials and methods

### A collection of genes with 5'UTR introns

NCBI's human Reference Gene Collection (RefSeq) [63] and the associated annotation table were downloaded from the UCSC genome browser [64], genome assembly of May 2004. The annotation table was parsed using the Galaxy website [65] (as of June 2007) to obtain 5UI coordinates. Specifically, we extracted all introns annotated to lie between two 5'UTR exons. Then we removed all the cases where another splice variant was present in the RefSeq collection such that any sequence within the intron was part of the coding region. Hence, all the introns in our final dataset were strictly present in the 5'UTR according to the annotation of RefSeq genes. 5'UTR exon coordinates were similarly retrieved as of June 2007. Recent studies suggest that nearly all human genes are alternatively spliced [13-15]. However, it is not clear what fraction of these events have biological significance as opposed to reflecting random noise associated with the less than perfect fidelity of the splicing machinery. Only when multiple independent sources of evidence support tissue-dependent alternative splicing can we be confident that these variants have real biological significance. Therefore, we used RefSeq transcripts, which are (unlike ESTs) manually curated and supported by multiple sources of evidence. For the comparisons between total lengths of 5UIs and the rest of the introns, we extracted coordinates of all non-5'UTR introns from the RefSeq annotation table (as of May 2009). A complete list of the genomic coordinates of 5UIs examined in this study is available as Additional file 1.

### Microarray data and analysis

The microarray data were downloaded from Gene Expression Atlas, which included expression data from 79 different tissues in humans [66]. We used the gcRMA-normalized data from the Affymetrix U133a and GNF1H arrays. Synergizer [42] was used to associate RefSeq genes with probe sets on the U133a array and custom Perl v5.8.8 scripts were used to parse the GNF1H annotation table (available on the Gene Expression Atlas website). The resulting correspondences of RefSeq IDs to probe sets on the GNF1H and U133a microarrays were merged to obtain a final mapping. Where multiple probe sets corresponded to a single RefSeq ID, the arithmetic mean of the expression values of all the probes was used to obtain a representative expression level for that RefSeq ID in each tissue. A single region of the genome can correspond to more than one RefSeq ID due to alternative splice variants and/or alternative promoters, and there were cases of a single probe set corresponding to multiple RefSeq IDs. To avoid overweighting such regions, we removed RefSeq IDs such that there were no duplicates. The representative RefSeq ID from each such probe set was chosen uniformly at random. For each gene with a 5UI, we calculated the mean expression level across all tissues and divided the genes into three groups with respect to total 5'UTR intronic length: short, 0 to 25%; intermediate, 25 to 75%; long, 75 to 100% in length. All expression analysis was performed using the R software package v2.6.0. In addition, the 'hexbin' [67] and 'zoo' [68] packages for the R platform were used.

### Functional enrichment of Gene Ontology categories

GoSTAT [41] and FuncAssociate [40] were used for functional trend analysis. We restricted the space of genes to all genes in the RefSeq collection because we used annotations in this collection to determine the set of genes with 5UIs. We used the RefSeq IDs as input for analysis with both programs. FuncAssociate uses Synergizer [42] to resolve the synonyms using Ensembl as the authority. To quantify the effect size, all the statistically significant GO categories that are enriched in the genes with introns are sorted according to their log_10 _odds ratio. All reported log odds ratios were obtained from FuncAssociate. Similar results were obtained using GoSTAT (data not shown).

### Comparative genomic analysis of non-receptor tyrosine kinases

To study the evolution of 5UI presence and length among NRTKs, we first identified orthologs of human NRTKs in the mouse and rat genomes. We used NCBI's Homologene Release 64 [69] (as of September 2009) to identify 'true' orthologous genes. Based on a recent evaluation of different approaches, Homologene showed greater specificity than other comparable orthology sources for the purposes of detailed phylogenetic and functional analysis [70]. We extracted the corresponding RefSeq IDs for each of the human NRTKs, and their mouse and rat orthologs. Then, we downloaded the RefSeq annotation tables for current genome builds (hg19, mm9, and rn4; as of September 2009) and used these annotations to determine 5UI lengths. All statistical analyses were performed using R software package v2.6.0. The raw data used in this analysis of human NRTKs are provided in Additional file 2.

### Motif discovery

The coordinates for the non-receptor tyrosine kinase genes that harbor introns were converted to human genome build hg18 using the LiftOver utility tool obtained from the UCSC Genome Browser website [71]. If there were known alternative splice variants in the RefSeq database, the longest intron was used for motif discovery purposes. Multiple alignment blocks for the human, mouse, and rat genomes (builds hg18, mm8, and rn4, respectively) were extracted from the 17-way multiZ alignment at the UCSC Genome Browser. These alignment blocks were merged using the Stitch MAF blocks utility on the Galaxy website [65] to obtain a final alignment of the human non-receptor tyrosine kinases to the mouse and rat orthologs. We obtained alignments that covered more than 10% of the length of the 5UIs for 37 human NRTKs, and excluded the other five introns from the subsequent motif discovery steps.

PhyloGibbs v1.2 was used in motif finding [45,72]. Different phylogenetic trees were tested but they did not significantly affect the results (not shown); therefore, all the results we report here were generated using the (hg18:0.5,(mm8:0.8, rn4:0.9):0.6) phylogeny specified in Newick tree format. Both RNA and DNA motifs (that is, forward strand only and both strands, respectively) were searched and the intronic sequences were used to define the background nucleotide distribution of the region to account for differences in nucleotide composition of 5UIs. The resulting motifs were represented by position-specific scoring matrices. The STAMP [47] web site was used to find similar motifs in the TRANSFAC v11.3 database as well as in a comparative genomics study in humans [46]. Default parameters were used in all comparisons.

### Analysis of the total exonic/intronic length and 5PCIs

To determine the lengths and positions of various genomic features, we first compiled a list of all RefSeq IDs. A single ID can correspond to multiple transcripts either that are expressed from the same or different regions in the genome. Such IDs can be associated with different transcript structures, and are therefore removed from further analysis. RefSeq IDs corresponding to genes in the hypervariable hla-locus were similarly represented multiple times in the RefSeq collection. In these cases, only the version in the reference genome was retained for further analysis.

After these initial filters, we calculated total lengths of 5UIs, 5'UTR exons, and other introns for each remaining RefSeq transcript. The position of the first coding intron was determined using the coordinates of all introns from the RefSeq annotation table that was retrieved as of May 2009. There were multiple identifiers for different splice variants that were transcribed from the same genomic location in the RefSeq collection. To avoid any systematic biases, we compared three different approaches in selecting RefSeq transcripts for further analysis. First, we kept all transcripts regardless of how many were transcribed from a given loci. Second, we determined equivalence classes of RefSeq transcripts, such that two IDs were in the same set if their transcription intervals (from start to stop position) overlapped by more than 20 base pairs. Then, we randomly removed RefSeqs transcripts such that only a single representative transcript remained for each equivalence class. Third, exact duplicates with respect to the 5'UTR were removed. Specifically, if two or more RefSeq IDs had the exact same 5'UTR, a single identifier was selected as a representative for that particular region. Splice variants that differ in their 5'UTR were not removed because these provide additional information about the lengths of 5'UTR introns and exons. All three methods yielded similar results and led to identical conclusions. Therefore, only one representative method is shown in the figures. The third method conveys the most information when discussing total 5UI lengths and hence was used in Figure 1a. By contrast, considering one representative from each transcriptional unit is more relevant when analyzing the correlation between two genomic features. Hence, the second method was used for Figures 1c, d.

For the specific GO categories used in our analysis, all the genes in a given category were retrieved from the human GOA database [73]. The corresponding RefSeq identifiers were determined using the Synergizer software [42]. Total exonic length and intronic length were calculated for all these genes as described above.

## Abbreviations

5PCI: 5' proximal coding region intron; 5UI: 5'UTR intron; CV: coefficient of variation; EST: expressed sequence tag; GO: Gene Ontology; kb: kilobase; NRTK: non-receptor protein tyrosine kinase; PCC: Pearson Correlation Coefficient; uAUG: upstream AUG; UTR: untranslated region.

## Authors' contributions

CC carried out all analyses, designed the study and drafted the manuscript. AD contributed to the generation of the 5UI dataset, provided guidance with all the analyses and contributed to the writing of the manuscript. JCM participated in the design of the study. GFB helped with functional enrichment analysis and contributed to the writing of the manuscript. FPR conceived and supervised the study, and contributed to the writing of the manuscript. All authors read and approved the final manuscript.

## Supplementary Material

Additional file 1 Complete list of RefSeq mRNA IDs that have 5'UTR introns. This file contains the genomic coordinates and RefSeq IDs for all transcripts with 5'UTR introns. '+'and '-' represent the forward and reverse strands, respectively. Click here for file

Additional file 2 Complete list of 5'UTR intron lengths for the human non-receptor tyrosine kinases and their orthologs in mouse and rat genomes. This file contains the RefSeq IDs and gene symbols for all human NRTKs and their mouse and rat orthologs. For all transcripts, 5'UTR intron lengths are given. Click here for file

Additional file 3 Overrepresented GO attributes for genes with 5'-proximal coding introns. This file contains the table of overrepresented GO attributes for genes with 5'-proximal coding introns. The methods and legend are the same as in Table 1. Click here for file

## References

[B1] Rodriguez-Trelles F Tarrio R Ayala FJ Origins and evolution of spliceosomal introns. Annu Rev Genet 2006 40 47 76 10.1146/annurev.genet.40.110405.090625 17094737

[B2] Roy SW Gilbert W The evolution of spliceosomal introns: patterns, puzzles and progress. Nat Rev Genet 2006 7 211 221 16485020 10.1038/nrg1807

[B3] Rogozin IB Wolf YI Sorokin AV Mirkin BG Koonin EV Remarkable interkingdom conservation of intron positions and massive, lineage-specific intron loss and gain in eukaryotic evolution. Curr Biol 2003 13 1512 1517 10.1016/S0960-9822(03)00558-X 12956953

[B4] Carmel L Rogozin IB Wolf YI Koonin EV Patterns of intron gain and conservation in eukaryotic genes. BMC Evol Biol 2007 7 192 10.1186/1471-2148-7-192 17935625 PMC2151770

[B5] Lynch M Conery JS The origins of genome complexity. Science 2003 302 1401 1404 10.1126/science.1089370 14631042

[B6] Comeron JM Kreitman M The correlation between intron length and recombination in drosophila. Dynamic equilibrium between mutational and selective forces. Genetics 2000 156 1175 1190 11063693 10.1093/genetics/156.3.1175 PMC1461334

[B7] Duret L Why do genes have introns? Recombination might add a new piece to the puzzle. Trends Genet 2001 17 172 175 10.1016/S0168-9525(01)02236-3 11275306

[B8] Niu DK Protecting exons from deleterious R-loops a potential advantage of having introns. Biol Direct 2007 2 11 10.1186/1745-6150-2-11 17459149 PMC1863416

[B9] Blencowe BJ Alternative splicing: new insights from global analyses. Cell 2006 126 37 47 10.1016/j.cell.2006.06.023 16839875

[B10] Xing Y Lee C Alternative splicing and RNA selection pressure - evolutionary consequences for eukaryotic genomes. Nat Rev Genet 2006 7 499 510 10.1038/nrg1896 16770337

[B11] Matlin AJ Clark F Smith CW Understanding alternative splicing: towards a cellular code. Nat Rev Mol Cell Biol 2005 6 386 398 10.1038/nrm1645 15956978

[B12] Johnson JM Castle J Garrett-Engele P Kan Z Loerch PM Armour CD Santos R Schadt EE Stoughton R Shoemaker DD Genome-wide survey of human alternative pre-mRNA splicing with exon junction microarrays. Science 2003 302 2141 2144 10.1126/science.1090100 14684825

[B13] Wang ET Sandberg R Luo S Khrebtukova I Zhang L Mayr C Kingsmore SF Schroth GP Burge CB Alternative isoform regulation in human tissue transcriptomes. Nature 2008 456 470 476 10.1038/nature07509 18978772 PMC2593745

[B14] Castle JC Zhang C Shah JK Kulkarni AV Kalsotra A Cooper TA Johnson JM Expression of 24,426 human alternative splicing events and predicted cis regulation in 48 tissues and cell lines. Nat Genet 2008 40 1416 1425 10.1038/ng.264 18978788 PMC3197713

[B15] Pan Q Shai O Lee LJ Frey BJ Blencowe BJ Deep surveying of alternative splicing complexity in the human transcriptome by high-throughput sequencing. Nat Genet 2008 40 1413 1415 10.1038/ng.259 18978789

[B16] Wang Z Burge CB Splicing regulation: from a parts list of regulatory elements to an integrated splicing code. RNA 2008 14 802 813 10.1261/rna.876308 18369186 PMC2327353

[B17] Sugnet CW Srinivasan K Clark TA O'Brien G Cline MS Wang H Williams A Kulp D Blume JE Haussler D Ares M Jr Unusual intron conservation near tissue-regulated exons found by splicing microarrays. PLoS Comput Biol 2006 2 e4 10.1371/journal.pcbi.0020004 16424921 PMC1331982

[B18] Pesole G Mignone F Gissi C Grillo G Licciulli F Liuni S Structural and functional features of eukaryotic mRNA untranslated regions. Gene 2001 276 73 81 11591473 10.1016/S0378-1119(01)00674-6

[B19] Hong X Scofield DG Lynch M Intron size, abundance, and distribution within untranslated regions of genes. Mol Biol Evol 2006 23 2392 2404 10.1093/molbev/msl111 16980575

[B20] Chang YF Imam JS Wilkinson MF The nonsense-mediated decay RNA surveillance pathway. Annu Rev Biochem 2007 76 51 74 10.1146/annurev.biochem.76.050106.093909 17352659

[B21] Maquat LE Nonsense-mediated mRNA decay in mammals. J Cell Sci 2005 118 1773 1776 10.1242/jcs.01701 15860725

[B22] Scofield DG Hong X Lynch M Position of the final intron in full-length transcripts: determined by NMD? Mol Biol Evol 2007 24 896 899 10.1093/molbev/msm010 17244600

[B23] Castillo-Davis CI Mekhedov SL Hartl DL Koonin EV Kondrashov FA Selection for short introns in highly expressed genes. Nat Genet 2002 31 415 418 12134150 10.1038/ng940

[B24] Urritia AO Hurst LD The signature of selection mediated by expression on human genes. Genome Res 2003 13 2260 2264 10.1101/gr.641103 12975314 PMC403694

[B25] Duret L Mouchiroud D Expression pattern and, surprisingly, gene length, shape codon usage in *Caenorhabditis, Drosophila*, and *Arabidopsis*. Proc Natl Acad Sci USA 1999 96 4482 4487 10.1073/pnas.96.8.4482 10200288 PMC16358

[B26] Ren X-Y Vorst O Fiers MWEJ Stiekema WJ Nap J-P In plants, highly expressed genes are the least compact. Trends Genet 2006 22 528 532 10.1016/j.tig.2006.08.008 16934358

[B27] Vinogradov AE 'Genome design' model and multicellular complexity: golden middle. Nucleic Acids Res 2006 34 5906 5914 10.1093/nar/gkl773 17062620 PMC1635334

[B28] Eisenberg E Levanon EY Human housekeeping genes are compact. Trends Genet 2003 19 362 366 10.1016/S0168-9525(03)00140-9 12850439

[B29] Vinogradov AE Compactness of human housekeeping genes: selection for economy or genomic design? Trends Genet 2004 20 248 253 10.1016/j.tig.2004.03.006 15109779

[B30] Vinogradov AE 'Genome design' model: evidence from conserved intronic sequence in human-mouse comparison. Genome Res 2006 16 347 354 10.1101/gr.4318206 16461636 PMC1415212

[B31] Chen J Sun M Hurst LD Carmichael GG Rowley JD Human antisense genes have unusually short introns: evidence for selection for rapid transcription. Trends Genet 2005 21 203 207 10.1016/j.tig.2005.02.003 15797613

[B32] Chen J Sun M Rowley JD Hurst LD The small introns of antisense genes are better explained by selection for rapid transcription than by 'genomic design'. Genetics 2005 171 2151 2155 10.1534/genetics.105.048066 16143605 PMC1456133

[B33] Chamary J-V Hurst LD Similar rates but different modes of sequence evolution in introns and at exonic silent sites in rodents evidence for selectively driven codon usage. Mol Biol Evol 2004 21 1014 1023 10.1093/molbev/msh087 15014158

[B34] Majewski J Ott J Distribution and characterization of regulatory elements in the human genome. Genome Res 2002 12 1827 1836 10.1101/gr.606402 12466286 PMC187578

[B35] Hughes SS Buckley CO Neafsey DE Complex selection on intron size in *Cryptococcus*. Mol Biol Evol 2008 25 247 253 10.1093/molbev/msm220 18171915

[B36] Roy SW Penny D Neafsey DE Evolutionary conservation of UTR intron boundaries in *Cryptococcus*. Mol Biol Evol 2007 24 1140 1148 10.1093/molbev/msm045 17374879

[B37] Eden E Brunak S Analysis and recognition of 5'UTR intron splice sites in human pre-mRNA. Nucleic Acids Res 2004 32 1131 1142 10.1093/nar/gkh273 14960723 PMC373407

[B38] Venter JC Adams MD Myers EW Li PW Mural RJ Sutton GG Smith HO Yandell M Evans CA Holt RA Gocayne JD Amanatides P Ballew RM Huson DH Wortman JR Zhang Q Kodira CD Zheng XH Chen L Skupski M Subramanian G Thomas PD Zhang J Gabor Miklos GL Nelson C Broder S Clark AG Nadeau J McKusick VA The sequence of the human genome. Science 2001 291 1304 1351 10.1126/science.1058040 11181995

[B39] Thattai M van Oudenaarden A Intrinsic noise in gene regulatory networks. Proc Natl Acad Sci USA 2001 98 8614 8619 10.1073/pnas.151588598 11438714 PMC37484

[B40] Berriz GF King OD Bryant B Sander C Roth FP Characterizing gene sets with FuncAssociate. Bioinformatics 2003 19 2502 2504 10.1093/bioinformatics/btg363 14668247

[B41] Beißbarth T Speed TP GOstat: find statistically overrepresented Gene Ontologies within a group of genes. Bioinformatics 2004 20 1464 1465 10.1093/bioinformatics/bth088 14962934

[B42] Berriz GF Roth FP The Synergizer service for translating gene, protein and other biological identifiers. Bioinformatics 2008 24 2272 2273 10.1093/bioinformatics/btn424 18697767 PMC2553440

[B43] Tsygankov AY Non-receptor protein tyrosine kinases. Front Biosci 2003 8 s595 635 10.2741/1106 12700079

[B44] King DC Taylor J Elnitski L Chiaromonte F Miller W Hardison RC Evaluation of regulatory potential and conservation scores for detecting cis-regulatory modules in aligned mammalian genome sequences. Genome Res 2005 15 1051 1060 10.1101/gr.3642605 16024817 PMC1182217

[B45] Siddharthan R Siggia ED Nimwegen Ev PhyloGibbs: aGibbs sampling motif finder that incorporates phylogeny. PloS Comput Biol 2005 1 e67 10.1371/journal.pcbi.0010067 16477324 PMC1309704

[B46] Xie X Lu J Kulbokas EJ Golub TR Mootha V Lindblad-Toh K Lander ES Kellis M Systematic discovery of regulatory motifs in human promoters and 30 UTRs by comparison of several mammals. Nature 2005 434 338 345 10.1038/nature03441 15735639 PMC2923337

[B47] Mahony S Benos PV STAMP: a web tool for exploring DNA-binding motif similarities. Nucleic Acids Res 2007 35 W253 258 10.1093/nar/gkm272 17478497 PMC1933206

[B48] Furger A O'Sullivan JM Binnie A Lee BA Proudfout NJ Promoter proximal splice sites enhance transcription. Genes Dev 2002 16 2792 2799 10.1101/gad.983602 12414732 PMC187476

[B49] Brinster RL Allen JM Behringer RR Gelinas RE Palmiter RD Introns increase transcriptional efficiency in transgenic mice. Proc Natl Acad Sci USA 1988 85 836 840 10.1073/pnas.85.3.836 3422466 PMC279650

[B50] Palmiter RD Sandgren EP Avarbock MR Allen DD Brinster RL Heterologous introns can enhance expression of transgenes in mice. Proc Natl Acad Sci USA 1991 88 478 482 10.1073/pnas.88.2.478 1988947 PMC50834

[B51] Jonsson JJ Foresman MD Wilson N McIvor RS Intron requirement for expression of the human purine nucleoside phosphorylase gene. Nucleic Acids Res 1992 20 3191 3198 10.1093/nar/20.12.3191 1620616 PMC312458

[B52] Le Hir H Nott A Moore MJ How introns influence and enhance eukaryotic gene expression. Trends Biochem Sci 2003 28 215 220 10.1016/S0968-0004(03)00052-5 12713906

[B53] Rose AB The effect of intron location on intron-mediated enhancement of gene expression in *Arabidopsis*. Plant J 2004 40 744 751 10.1111/j.1365-313X.2004.02247.x 15546357

[B54] Rose AB Elfersi T Parra G Korf I Promoter-proximal introns in *Arabidopsis thaliana *are enriched in dispersed signals that elevate gene expression. Plant Cell 2008 20 543 551 10.1105/tpc.107.057190 18319396 PMC2329928

[B55] Fong YW Zhou Q Stimulatory effect of splicing factors on transcriptional elongation. Nature 2001 414 929 933 10.1038/414929a 11780068

[B56] Chan RY Boudreau-Lariviere C Angus LM Mankal FA Jasmin BJ An intronic enhancer containing an N-box motif is required for synapse- and tissue-specific expression of the acetylcholinesterase gene in skeletal muscle fibers. Proc Natl Acad Sci USA 1999 96 4627 4632 10.1073/pnas.96.8.4627 10200313 PMC16383

[B57] Lu J Sivamani E Azhakanandam K Samadder P Li X Qu R Gene expression enhancement mediated by the 5' UTR intron of the rice rubi3 gene varied remarkably among tissues in transgenic rice plants. Mol Genet Genomics 2008 279 563 572 10.1007/s00438-008-0333-6 18320227

[B58] Rohrer J Conley ME Transcriptional regulatory elements within the first intron of Bruton's tyrosine kinase. Blood 1998 91 214 221 9414287

[B59] Smith L Post-transcriptional regulation of gene expression by alternative 5'-untranslated regions in carcinogenesis. Biochem Soc Trans 2008 36 708 711 10.1042/BST0360708 18631145

[B60] Rosenstiel P Huse K Franke A Hampe J Reichwald K Platzer C Roberts RG Mathew CG Platzer M Schreiber S Functional characterization of two novel 5' untranslated exons reveals a complex regulation of NOD2 protein expression. BMC Genomics 2007 8 472 10.1186/1471-2164-8-472 18096043 PMC2228316

[B61] Araud T Genolet R Jaquier-Gubler P Curran J Alternatively spliced isoforms of the human elk-1 mRNA within the 5' UTR implications for ELK-1 expression. Nucleic Acids Res 2007 35 4649 4663 10.1093/nar/gkm482 17591614 PMC1950554

[B62] Ma XM Yoon S-O Richardson CJ Julich K Blenis J SKAR links pre-mRNA splicing to mTOR/S6K1-mediated enhanced translation efficiency of spliced mRNAs. Cell 2008 133 303 313 10.1016/j.cell.2008.02.031 18423201

[B63] Pruitt KD Tatusova T Maglott DR NCBI reference sequences (RefSeq): a curated non-redundant sequence database of genomes, transcripts and proteins. Nucleic Acids Res 2007 35 D61 D65 10.1093/nar/gkl842 17130148 PMC1716718

[B64] Rhead B Karolchik D Kuhn RM Hinrichs AS Zweig AS Fujita PA Diekhans M Smith KE Rosenbloom KR Raney BJ Pohl A Pheasant M Meyer LR Learned K Hsu F Hillman-Jackson J Harte RA Giardine B Dreszer TR Clawson H Barber GP Haussler D Kent WJ The UCSC Genome Browser database: update 2010. Nucleic Acids Res 2010 38 D613 D619 10.1093/nar/gkp939 19906737 PMC2808870

[B65] Giardine B Riemer C Hardison RC Burhans R Elnitski L Shah P Zhang Y Blankenberg D Albert I Taylor J Miller W Kent WJ Nekrutenko A Galaxy: a platform for interactive large-scale genome analysis. Genome Res 2005 15 1451 1455 10.1101/gr.4086505 16169926 PMC1240089

[B66] Su AI Wiltshire T Batalov S Lapp H Ching KA Block D Zhang J Soden R Hayakawa M Kreiman G Cooke MP Walker JR Hogenesch JB A gene atlas of the mouse and human protein-encoding transcriptomes. Proc Natl Acad Sci USA 2004 101 6062 6067 10.1073/pnas.0400782101 15075390 PMC395923

[B67] hexbin: Hexagonal Binning Routines. R package version 1.18.0 http://www.bioconductor.org/packages/bioc/html/hexbin.html

[B68] Zeileis A Grothendieck G zoo: S3 Infrastructure for Regular and Irregular Time Series. J Stat Software 2005 14 1 27

[B69] HomoloGene http://www.ncbi.nlm.nih.gov/homologene

[B70] Altenhoff AM Dessimoz C Phylogenetic and functional assessment of orthologs inference projects and methods. PLoS Comput Biol 2009 5 e1000262 10.1371/journal.pcbi.1000262 19148271 PMC2612752

[B71] UCSC Genome Browser LiftOver Utility http://genome.ucsc.edu/cgi-bin/hgLiftOver

[B72] Siddharthan R Nimwegen E Detecting regulatory sites using PhyloGibbs. Methods in Molecular Biology 2007 Bergman NH: Humana Press 382 402 10.1007/978-1-59745-514-5_24 17993687

[B73] Barrell D Dimmer E Huntley RP Binns D O'Donovan C Apweiler R The GOA database in 2009 - an integrated Gene Ontology Annotation resource. Nucleic Acids Res 2009 37 D396 D403 10.1093/nar/gkn803 18957448 PMC2686469

[B74] Sandelin A Wasserman WW Constrained binding site diversity within families of transcription factors enhances pattern discovery bioinformatics. J Mol Biol 2004 338 207 215 10.1016/j.jmb.2004.02.048 15066426

