# Supplementary material for: Genome-wide functional analysis of human 5' untranslated region introns
Source: Genome Biol. 2010 Mar 11;11(3):R29. doi: 10.1186/gb-2010-11-3-r29 (PMC2864569; doi:10.1186/gb-2010-11-3-r29)
Supplement: Additional file 3 — Overrepresented GO attributes for genes with 5'-proximal coding introns. This file contains the table of overrepresented GO attributes for genes with 5'-proximal coding introns. The methods and legend are the same as in Table 1. [file gb-2010-11-3-r29-S3.PDF]

Rank	N	X	LOD	P	P-adj		GO Attribute
1	14	15	1.416	1.3e-07	<0.0001	GO:0032393:	MHC class I receptor activity
2	24	28	1.168	1.5e-10	<0.0001	GO:0002474:	antigen processing and presentation of peptide antigen via MHC class I
3	39	46	1.155	4.3e-16	<0.0001	GO:0042612:	MHC class I protein complex
4	17	20	1.130	1.1e-07	<0.0001	GO:0022627:	cytosolic small ribosomal subunit
5	26	31	1.115	6.5e-11	<0.0001	GO:0048002:	antigen processing and presentation of peptide antigen
6	11	13	1.093	2.5e-05	0.0218	GO:0005833:	hemoglobin complex
7	14	17	1.048	3.3e-06	0.0032	GO:0004364:	glutathione transferase activity
8	19	24	0.981	1.6e-07	<0.0001	GO:0022625:	cytosolic large ribosomal subunit
9	35	46	0.923	6.1e-12	<0.0001	GO:0022626:	cytosolic ribosome
10	16	22	0.836	1.1e-05	0.0094	GO:0009109:	coenzyme catabolic process
11	16	22	0.836	1.1e-05	0.0094	GO:0015145:	monosaccharide transmembrane transporter activity
12	16	22	0.836	1.1e-05	0.0094	GO:0015149:	hexose transmembrane transporter activity
13	15	21	0.808	2.9e-05	0.0287	GO:0005355:	glucose transmembrane transporter activity
14	15	21	0.808	2.9e-05	0.0287	GO:0006099:	tricarboxylic acid cycle
15	15	21	0.808	2.9e-05	0.0287	GO:0046356:	acetyl-CoA catabolic process
16	48	69	0.788	2.2e-13	<0.0001	GO:0042611:	MHC protein complex
17	17	25	0.745	2.3e-05	0.0181	GO:0051187:	cofactor catabolic process
18	27	40	0.741	1.1e-07	<0.0001	GO:0015934:	large ribosomal subunit
19	96	150	0.687	2.6e-21	<0.0001	GO:0004252:	serine-type endopeptidase activity
20	19	30	0.660	3.5e-05	0.0348	GO:0009060:	aerobic respiration
21	73	117	0.654	1.2e-15	<0.0001	GO:0044445:	cytosolic part
22	103	168	0.638	1e-20	<0.0001	GO:0008236:	serine-type peptidase activity
23	103	168	0.638	1e-20	<0.0001	GO:0017171:	serine hydrolase activity
24	85	139	0.633	3.2e-17	<0.0001	GO:0003735:	structural constituent of ribosome
25	47	77	0.626	4.2e-10	<0.0001	GO:0033279:	ribosomal subunit
26	29	48	0.612	1.3e-06	0.0008	GO:0015295:	solute:hydrogen symporter activity
27	27	45	0.604	3.6e-06	0.0033	GO:0005351:	sugar:hydrogen symporter activity
28	27	46	0.581	6.5e-06	0.0047	GO:0015144:	carbohydrate transmembrane transporter activity
29	27	46	0.581	6.5e-06	0.0047	GO:0051119:	sugar transmembrane transporter activity
30	104	180	0.574	3.2e-18	<0.0001	GO:0005840:	ribosome
31	53	94	0.544	2e-09	<0.0001	GO:0019882:	antigen processing and presentation
32	30	56	0.493	2.4e-05	0.0191	GO:0051258:	protein polymerization
33	40	76	0.478	2.1e-06	0.0014	GO:0007018:	microtubule-based movement
34	44	85	0.463	1.2e-06	0.0007	GO:0030705:	cytoskeleton-dependent intracellular transport
35	168	346	0.418	6.1e-18	<0.0001	GO:0030529:	ribonucleoprotein complex
36	159	358	0.343	8e-13	<0.0001	GO:0004175:	endopeptidase activity
37	126	293	0.316	2.3e-09	<0.0001	GO:0006412:	translation
38	206	504	0.282	6.6e-12	<0.0001	GO:0008233:	peptidase activity
39	132	321	0.282	2.8e-08	<0.0001	GO:0005829:	cytosol
40	184	455	0.272	2.6e-10	<0.0001	GO:0006955:	immune response
41	228	579	0.255	3.8e-11	<0.0001	GO:0006508:	proteolysis
42	585	1598	0.222	3.8e-19	<0.0001	GO:0032991:	macromolecular complex
43	212	563	0.222	1.6e-08	<0.0001	GO:0002376:	immune system process
44	132	357	0.205	2.4e-05	0.0187	GO:0006952:	defense response
45	158	441	0.184	2.8e-05	0.0233	GO:0003723:	RNA binding
46	430	1252	0.166	1.8e-09	<0.0001	GO:0005576:	extracellular region
47	441	1302	0.156	8.5e-09	<0.0001	GO:0043234:	protein complex
48	673	2171	0.100	5.6e-06	0.0045	GO:0044267:	cellular protein metabolic process
49	710	2307	0.096	8.6e-06	0.0077	GO:0019538:	protein metabolic process

50	687	2240	0.092	2.1e-05	0.0172	GO:0044260:	cellular macromolecule metabolic process
